# How achievement motive enactment shapes daily flow experience and work engagement: The interplay of personality systems

**DOI:** 10.1007/s11031-021-09894-2

**Published:** 2021-06-09

**Authors:** Jan Digutsch, Stefan Diestel

**Affiliations:** 1 Flexible Control of Behaviour, Leibniz Research Centre for Working Environment and Human Factors at the Technical University Dortmund, Ardeystr. 67, 44139 Dortmund, Germany; 2 Schumpeter School of Business and Economics, Faculty of Economy, University of Wuppertal, Wuppertal, Germany

**Keywords:** PSI theory, Achievement motive enactment, Flow experience, Work engagement, Role clarity

## Abstract

In the present study, we examined how different forms of achievement motive interact to predict daily flow experience and work engagement. In particular, we conducted two diary studies to examine the main and interaction effects of motive enactment via extension memory (a macrosystem that enables holistic and experience-based information processing) and via the object recognition system (an alert-oriented macrosystem). In study 1, in line with personality systems interaction (PSI) theory, we found that motive enactment via extension memory fosters both day-specific flow and work engagement, whereas the conjunction of both forms of motive enactment has beneficial effects on flow and work engagement (two-way interaction). In study 2, we found that role clarity moderates the interaction of the two forms of enactment, indicating that the two-way interaction occurs when role clarity is low. Our results imply that the interplay of different dispositional forms of achievement motive enactment shapes how employees experience flow and engagement.

## Introduction

In different occupational and organizational settings, flow experience and work engagement have been repeatedly linked with psychological well-being (e.g., Peifer & Engeser, 2021; Rivkin et al., 2018) and job performance (e.g., Christian et al., 2011). Flow refers to peak experiences of energized motivation when people take on a task and is characterized by high involvement in an activity, a high sense of control, and a decelerated sense of time (Csikszentmihalyi & LeFevre, 1989; Csikszentmihalyi et al., 2005; Rivkin et al., 2018). People engage in flow experience when they perceive a balance between their skills and the demands of a given task (Csikszentmihalyi, 1975/2000, 1990; Csikszentmihalyi & Larson, 1987; Csikszentmihalyi & LeFevre, 1989; Nakamura & Csikszentmihalyi, 2002). Work engagement is defined as a positive, fulfilling, work-related state of mind characterized by absorption, vigor, and dedication (Schaufeli et al., 2002). By experiencing this motivational state, people fully engage themselves in a difficult task for the sake of the activity itself (Baumann & Scheffer, 2011). Both flow experience and work engagement show conceptual overlap but have differences in their duration (a peak experience vs. an ongoing state, respectively; Hallberg & Schaufeli, 2006; Sonnentag, 2003) and scope (Csikszentmihalyi et al., 2005; Schaufeli et al., 2002).

Although most research has primarily focused on fluctuating states of flow and work engagement, only a few studies have explored more dispositional predictors of stable patterns in both outcomes over time. For example, Csikszentmihalyi (1990) introduced the concept of the autotelic personality, which describes individuals who tend to position themselves in situations that enable frequent experiences of flow states (Asakawa, 2014). High autotelic personality scores are positively related to the need for achievement (Csikszentmihalyi et al., 1993) and a stable motivational disposition, which is characterized by a recurrent preference for affectively rewarding experiences related to improving one's performance (Atkinson, 1957; McClelland, 1985). Past research has demonstrated that such motivational dispositions drive goal-directed behavior (e.g., Beckmann & Heckhausen, 2008). We argue that workplaces provide many situational cues that activate the achievement motives of employees, such as highly challenging tasks, goals, feedback, and performance systems (see goal-setting theory, Locke & Latham, 1990), thereby facilitating employees’ flow experience (Csikszentmihalyi, 1990; Csikszentmihalyi et al., 2005). In line with our argument, Baumann and Scheffer (2010, 2011) investigated whether the stable need to seek and master difficulties, as intrinsic components of achievement motive, is related to flow experience. The results showed that individuals who actively seek involvement in challenging tasks and enjoy the process of mastering these challenges are more likely to experience flow. These results were in line with the dialectical principle inherent in autotelic experiences, that is, the simultaneous presence of two opposing processes: differentiation and integration (Csikszentmihalyi et al., 1993).

Although other scholars have repeatedly explored personality as an antecedent of flow and work engagement, past research has failed to provide nuanced evidence on how and when personality traits predict both outcomes. The weak focus on personality traits is surprising given the substantial between-person variance (in contrast to within-person variance) in both flow experience and work engagement, suggesting that their respective levels are considerably influenced by personality traits and job characteristics (e.g., Diestel et al., 2015; Rivkin et al., 2018). Other personality approaches have usually focused on either motivation (e.g., achievement motive; Engeser & Rheinberg, 2008) or volition (e.g., action orientation: Baumann et al., 2016; Keller & Bless, 2008; Wojdylo et al., 2014), contributing to addressing the question of what people strive for or how they strive for it.

To shed light on both perspectives simultaneously, our study not only focuses on achievement motive itself but also on two components of achievement motive *enactment*. The conceptual difference between these components is grounded in personality systems interaction theory (PSI theory; Kuhl, 2000). In summary, PSI theory distinguishes between four macrosystems (intuitive behavior control, object recognition, extension memory, and intention memory) that have distinctive modulative functions for information processing and the regulation of behavioral and decision processes. The intuitive behavior system involves unconscious procedural knowledge about engagement in specific behavioral patterns such as sensorimotor and behavioral processes. The object recognition system focuses on threats, problems, and other stressful aspects of situations and thus acts as a kind of alarm system. Extension memory (also referred to as the integrated self, Kuhl et al., 2015) is based on parallel-distributed and holistic processing and integrates experiences in coherent and sense-making self-related representations by integrating environmental factors with personal values and experiences. Intention memory is based on sequential analytical processing and facilitates the formation of intentions, action planning, and goal setting.

Achievement motive can be enacted via these macrosystems, resulting in dispositional cognitive styles that shape the way individuals strive for motive-related incentives in their environments. While achievement motive enactment via the object recognition system causes individuals to focus on isolated negative experiences through which they become more alert and sensitive to discrepancies in their need for achievement, achievement motive enactment via extension memory is driven by parallel instead of sequential and integrative processing of goal-relevant information and situational cues (Baumann & Kuhl, 2002). In occupational contexts, achievement motive enactment via the object recognition system involves the perception of a negative event (e.g., critical feedback from a supervisor) as a single experience (“object”), which requires tolerance of frustrating experiences. Enactment via extension memory turns those vulnerabilities into emotional strength by integrating isolated experiences into one’s autobiographical network and overcoming negative affect (Kuhl et al., 2015). If one macrosystem works without the other, there are either no new learning experiences (low object recognition and high extension memory), or the experiences cannot be put into a broader context (high object recognition and low extension memory). Several studies have provided empirical support for this dynamic interplay of macrosystems (e.g. Bledow et al., 2011; Yang et al., 2016). Bledow et al. (2011) found that the shift between macrosystems results in disproportionally high work engagement, indicating that work engagement emerges from a dynamic interplay of affect that initiates those macrosystems.

Going beyond existing knowledge about the interactions of different affects, we predict that two macrosystems should interact in regulating achievement motive enactment, thereby fostering flow experience and work engagement. In light of the finding that the impact of traits on motivational processes is contingent upon job conditions (van den Berg & Feij, 2003), we also examine whether role clarity moderates the interaction effect of two forms of achievement motive enactment on day-specific work engagement and flow experience. Role clarity is defined as the degree to which employees receive clear and consistent information about their tasks and goals and other relevant job conditions (Kahn et al., 1964; Kauppila, 2014; Rizzo et al., 1970). Kahn (1990) identified role clarity as an antecedent of work engagement given its function as a resource, as clarity regarding work methods and processes is necessary for task completion and goal achievement (Bliese & Castro, 2000; Gillet et al., 2016) and has been positively linked with self-efficacy, performance, commitment and work engagement (e.g., Chen & Bliese, 2002; Halbesleben, 2010; Örtqvist & Wincent, 2006; Seppälä et al., 2015; Venz et al., 2018; Whitaker et al., 2007). In line with the plasticity hypothesis (Brockner, 1983), which states that individuals are influenced by environmental factors to different degrees according to their individual characteristics, we expect role clarity to moderate the interaction between achievement motive enactment via the object recognition system and achievement motive enactment via extension memory. When employees perceive high role clarity, motive enactment should be less relevant for flow and work engagement since a match between skills and task requirements is provided by a clear task structure. In contrast, when role clarity is low, dispositional antecedents (i.e., achievement motive) have a stronger influence on flow and work engagement. Individuals differ in the way they identify and engage with their tasks and experience flow during task completion depending on their personalities (i.e., how they enact their achievement motive).

In our study, we seek to make four contributions to the literature on flow and work engagement. First, we shed light on the interplay of motivation and volition by going beyond existing knowledge about the impact of achievement motive on motivational states at work. We identify different dispositional tendencies in achievement motive enactment, thereby explaining how high levels of achievement motive facilitate flow and work engagement over the course of several working days. In particular, we consider not only the main effects of traits (e.g., achievement motive) but also the interaction effects of two forms of achievement motive enactment. In doing so, we seek to extend the scholarly understanding of the impact of achievement motive on flow and work engagement by using two different but complementary forms of achievement motive enactment, which form the mechanistic basis of general motive strength.

Second, by employing a daily diary study, we explicitly take temporal dynamics in flow and work engagement into account. In particular, our research design allows us to explore lagged main and interaction effects of personality systems on motivational states and thus control for temporal fluctuations and different situational contingencies over time (see Ohly et al., 2010 for an overview).

Third, we propose achievement motive enactment as a dispositional antecedent of flow and work engagement. As noted by Baumann and Scheffer (2011), stable dispositions and their interplay with each other constitute a neglected domain of research. Moreover, dispositional individual differences shape people’s tendencies regarding the frequency of and ability for flow and work engagement (Csikszentmihalyi et al., 1993; Haworth et al., 1997; Kahn, 1990; Keller & Bless, 2008; Keller & Blomann, 2008).

Fourth, we extend the research on the interplay between job characteristics and achievement motive enactment on work engagement and flow experience by introducing role clarity, which has previously been identified as an antecedent of work engagement, as a moderating variable (Kahn, 1990). Building upon existing knowledge on interactions between personality and working environment, we apply the plasticity hypothesis (Brockner, 1983) on the role of achievement motive in developing flow and work engagement.

In the following, we elaborate on how two forms of achievement motive enactment differentially relate to flow and work engagement. Then, we derive hypotheses about the main and interaction effects of the two forms of enactment on flow experiences and work engagement. We test these predictions through a daily diary study over five consecutive working days. In addition, we elaborate on the impact of role clarity on the hypothesized two-way interaction by analyzing a three-way interaction in a second diary study.

## Day-specific flow and work engagement and achievement motive enactment

As stated before, a balance between individual skills and job demands is a prerequisite for experiencing flow and engaging in certain tasks at work (e.g., Csikszentmihalyi, 1975/2000, 1990). When achievement motive is enacted via extension memory, individuals can access their extensive semantic network that stores integrative experiences and fosters high-level intuitive information processing. This cautious, flexible, and holistic processing enables individuals to access implicit self-representations (i.e., prior memories, values, needs, experiences, and motives). Difficulties are likely to be perceived as challenges rather than potential hindrances. When achievement motive is not enacted via extension memory, individuals are at risk of pursuing goals and following tasks that are not congruent with their personalities. Past research has demonstrated that the congruence of implicit self-representations is essential for flow experience (e.g., Schüler et al., 2014). Consequently, we expect that the enactment of achievement motives via the integrated self facilitates day-specific flow and work engagement over time.

### H1:

Achievement motive enactment via extension memory is positively related to (a) day-specific work engagement and (b) day-specific flow experience.

As the opposite system to extension memory, the object recognition system primarily implies alert-driven attention regulation (Kuhl, 2000). Such forms of regulation involve shifting an individual’s attentional focus to threats, problems, or other stressful aspects of situations to detect discrepancies between the situation and the person’s wishes or expectancies (Koole et al., 2019). Several experimental studies have provided convergent empirical evidence that access to implicit self-representations is reduced when the object recognition system is activated (Baumann & Kuhl, 2002; Kazén et al., 2003). As a result, people are less able to reduce negative affect (e.g., Koole & Jostmann, 2004).

If individuals enact their achievement motive via the object recognition system, they become more alert and sensitive to discrepancies in external stimuli and internal “objects” of experience (Kuhl et al., 2006). The object recognition system is activated primarily in cases of an imbalance and hence causes employees to enact their achievement motive in a way that impedes flow and work engagement. An imbalance occurs when the individual’s skills and the challenge of the task are not matched, either because the individual’s skills exceed the challenge (the individual feels bored) or the challenge exceeds the individual’s skills (the individual feels anxious). Whereas awareness of this imbalance might be beneficial for goal monitoring, as it is sensitive to deviations from expectations, standards, or goal objectives, the negative affect associated with such an imbalance can impair flow and work engagement. Therefore, we expect achievement motive enactment via the object recognition system to hinder flow and work engagement.

### H2:

Achievement motive enactment via the object recognition system is negatively related to (a) day-specific work engagement and (b) day-specific flow experience.

## Dynamic interplay of achievement motive enactment via the object recognition system and extension memory

According to PSI theory, not only is human personality characterized by dispositional tendencies in one of the four macrosystems, but its functioning is primarily driven by interactions between all four macrosystems. A main task for individuals is to achieve personal growth, meaning that an individual is open to new (i.e., unexpected or undesired) information that can be integrated into existing networks of autobiographical knowledge (Koole et al., 2019). A prerequisite for personal growth is the ability to flexibly switch between the object recognition system and extension memory. For instance, the activation of extension memory downregulates activities in the object recognition system, thereby allowing individuals to use parallel processing of both current and past personal experiences to integrate new experiences into existing networks of knowledge, experiences, and values (Baumann & Kuhl, 2002). If the opposite is the case (i.e., a strong activation of the object recognition system paired with a weak activation of extension memory), individuals become more alert and open to undesirable experiences, and they focus on isolated stimuli and objects (Yang et al., 2016).

As a result of the interaction of both forms of motive enactment, individuals will most notably engage in flow and work engagement if they are able to simultaneously access both macrosystems when striving to achieve their goals (Kuhl, 2000, 2001). According to Bledow et al. (2011), the initial negative relation of negative events to day-specific work engagement can become motivational potential, which is manifested as disproportionally high work engagement. That is, if individuals can focus on the problematic situation (object recognition system) and integrate those experiences into the broad semantic network of the self (extension memory), the combination of both forms of enactment can enable individuals to develop and extend their self, which, in turn, fosters flow and work engagement (Bledow et al., 2011; Yang et al., 2016). This dynamic interplay of both macrosystems is called self-development, an internal mechanism that describes the ability to regulate negative affect, which enables the integration of new experiences into extended networks of individual prior experiences (Kuhl et al., 2006). Self-development has also been identified as a prerequisite for an integrated self, which is often used as a descriptive term that indicates significant behavioral achievements (Kuhl et al., 2006). In support of this line of reasoning, Bledow et al. (2011) and Yang et al. (2016) found that downshifts in negative affect enhanced the positive relations of upshifts in positive affect with high work engagement and organizational citizenship behavior.

An employee who strongly tends to enact his/her achievement motive only via the object recognition system is likely to experience a negative affective state when he/she receives negative feedback from his/her supervisor. This negative feedback impairs the affectively rewarding experiences related to improving one's performance that are necessary for flow and work engagement for individuals with high achievement motive. Without the opportunity to learn from this feedback, the person is likely to remain in a state that hinders flow and work engagement. However, if the person can integrate those experiences to learn and self-develop based on the negative feedback (i.e., to integrate the experiences into extension memory), flow and work engagement should increase.

### H3:

Achievement motive enactment via extension memory interacts with achievement motive enactment via the object recognition system in predicting (a) work engagement and (b) flow experience such that when achievement motive enactment via extension memory is high, the relations of achievement motive enactment via the object recognition system to both outcomes are positive, whereas when achievement motive enactment via extension memory is low, the relations of achievement motive enactment via the object recognition system to both outcomes are negative.

## Study 1

### Methods

#### Sample and study design

To test our proposed hypotheses, we conducted a daily diary study, as this study design offers several methodological benefits for the analysis of the relation between person-level predictors and day-specific outcomes. Both flow experience and work engagement exhibited high levels of within-person variance in prior studies, thereby calling for diary studies, which allow for the thorough separation of different sources of variance (Rivkin et al., 2018; Sonnentag et al., 2010). In addition, between-person variance in day-specific variables is contingent on person-specific factors (i.e., interindividual dispositions; Diestel et al., 2015; Kühnel et al., 2012). The repeated measures within the diary study ensured the reliable measurement of temporal fluctuation in both outcome variables at the individual level. Thus, our longitudinal design allowed for the prediction of states of flow and work engagement. Third, the use of multiple measures over the course of several workdays also helped reduce common-method variance caused by the sole use of self-report measures (Podsakoff et al., 2003).

The participants (German employees primarily from the service sector with regular contact with customers, clients, patients, or other individuals at work) were recruited via the e-mail distribution lists of several universities and social networks such as LinkedIn, XING, and Facebook. Overall, 62 employees (44.19% part-time) with an average age of 33.61 years (*SD* = 13.99) were included in this study. A total of 59.68% of the sample was women. On average, the participants completed 71.94% of the day-specific questionnaires. In advance of the day-specific measurements, the participants responded to a general questionnaire that assessed biographical variables and person-level constructs (e.g., achievement motive enactment). Over five consecutive workdays, the participants received e-mails to answer day-specific questions about work engagement and flow experience two hours after the end of work. After the participants received the e-mails, the surveys were accessible for 4 h, with a reminder being sent after two hours. On weekends and public holidays, the diary study was suspended and continued the next regular working day. There was no drop-out between the general questionnaire and the day-specific measurements.

In line with Meier et al. (2013), we tested the deterioration of compliance over time by examining whether the day of study (ranging from 1 to 5) was related to missing data (0 = *no missing data*; 1 = *missing data*). The data suggest that the day of the study was unrelated to missing data (*r* = 0.02, *n.s.*), indicating that compliance did not deteriorate over time.

#### Measures

##### Achievement motive enactment

The Motive Enactment Test (MET; Kuhl, 1999; Kuhl & Henseler, 2003) was used to measure achievement motive enactment via both extension memory (e.g., “I can thoroughly identify myself with most of the tasks I assume” and “When I think about my previous achievements, I feel very good”) and the object recognition system (e.g., “No matter how good my performance is, I still see critical aspects” and “A bad performance can truly pull me down”). The items were scored on a four-point rating scale ranging from 0 (“does not apply”) to 3 (“completely applies”).

##### Day-specific work engagement

The German version of the Work Engagement Scale (Schaufeli et al., 2006) was used to measure day-specific work engagement. The scale has three subscales, namely, vitality, dedication, and absorption, and a total of nine items (e.g., “In this moment, I feel bursting with energy”). The responses are provided on a seven-point rating scale from 0 (“does not apply”) to 6 (“completely applies”).

##### Day-specific flow experience

Four items from Rheinberg et al. (2003) were used to measure day-specific flow experience. The items were easily adapted for the content to refer specifically to the working day (e.g., “Today at work, I was focused entirely on what I was doing”). The participants were asked to provide their answers to the items on a seven-point rating scale from 1 (“not at all”) to 7 (“completely”).

#### Construct validity of the day-level variables

We conducted multilevel confirmatory factor analyses (MCFAs) to test the divergent validity of the day-level variables work engagement and flow experience. First, we tested a two-factor measurement model including the two variables as distinct factors. The fit indices for this model indicated a satisfactory fit: χ^2^ (128) = 211.38, *p* < 0.01, root mean square error of approximation (RMSEA) = 0.054, comparative fit index (CFI) = 0.957, standardized root mean square residual within-person/between-person (SRMRw/SRMRb) = 0.044/0.046. In contrast, a model integrating the two day-level variables into one common factor performed worse (χ^2^ (130) = 319.85, *p* < 0.01, RMSEA = 0.081, CFI = 0.903, SRMRw/SRMRb = 0.061/0.061).

Taken together, the results of the conducted MCFAs suggest that the two day-level variables work engagement and flow experience represented distinct constructs.

### Analytical procedure

Hierarchical linear modeling takes into account the independence of nested data since an interaction between the two levels is possible (Hox, 2002). All specifications and parameters were calculated using the program MLwiN (Rasbash et al., 2019). The null model contained only the intercept. In model 1, the person-level variables gender and age were added. In model 2, the person-level variables achievement motive enactment via the object recognition system and achievement motive enactment via extension memory were included as the main predictors. Model 3 included the interaction between the two predictors added in model 2. The person-level variables achievement motive enactment via the object recognition system and achievement motive enactment via extension memory were centered around the grand mean (Enders & Tofighi, 2007) to reduce the risk of multicollinearity in the analysis of the hypothesized interaction effect.

## Results

Appendix Table 1 displays the descriptive statistics, internal consistencies (Cronbach’s alphas) and correlations among the study variables. Before testing our hypotheses, we examined the within-person (level 1) variance of work engagement and flow experience. The proportion of within-person variance was 41.3% for work engagement and 52.1% for flow experience, indicating substantial level 2 variance in both dependent variables. These high levels of day-specific fluctuation justified the application of multilevel modeling.

**Table 1:** Means, standard deviations, internal consistencies (Cronbach’s alphas) and intercorrelations of the study variables

Variable	1	2	3	4	5	6
1. Work engagement	(0.95)	**0.79**				
2. Flow experience	**0.80**	(0.87)				
3. AME-ORS^a^	− 0.05	− 0.05	(0.79)			
4. AME-EM^b^	**0.36**	0.21	− 0.20	(0.68)		
5. Age	**0.37**	**0.50**	0.09	0.01	–	
6. Gender^c^	0.15	0.22	− 0.10	− 0.22	0.25	–
M	4.17	3.89	1.73	3.05	33.61	1.40
SD	1.18	1.35	0.59	0.52	13.99	0.49

The Cronbach’s alpha for day-level variables is the mean internal consistency averaged over all measurement days. Correlations below the diagonal are person-level correlations (*N* = 62); those above the diagonal are day-level correlations (*N* = 223). Numbers in bold indicate *p* < .05

^a^Achievement motive enactment via the object recognition system

^b^Achievement motive enactment via extension memory

^c^Gender (1 = female, 2 = male)

### Hypothesis testing

According to Hypothesis 1, we predicted that achievement motive enactment via extension memory would be positively related to (a) day-specific work engagement and (b) day-specific flow experience. In line with this prediction, the multilevel estimates (see Appendix Table 2) revealed that achievement motive enactment via extension memory was significantly positively related to both work engagement (γ = 0.42, SE = 0.13, *p* < 0.01) and flow experience (γ = 0.34, SE = 0.15, *p* < 0.01). In addition, Model 2 showed an improved fit compared with that of Model 1, as indicated by the difference in the log likelihood ratios for work engagement (Δ – 2 × log = 11.0, df = 2, *p* < 0.01) and flow experience (Δ − 2 × log = 6.6, df = 2, *p* < 0.05).

**Table 2:** multilevel estimates for the prediction of work engagement and flow experience

Effects	Work engagement								Flow experience							
	Null model		Model 1		Model 2		Model 3		Null model		Model 1		Model 2		Model 3	
	γ	SE	γ	SE	γ	SE	γ	SE	γ	SE	γ	SE	γ	SE	γ	SE
Fixed effects																
Intercept	4.30**	(0.14)	4.29**	(0.14)	4.28**	(0.12)	4.34**	(0.12)	4.14**	(0.15)	4.12**	(0.14)	4.12**	(0.14)	4.19**	(0.13)
Age			0.24	(0.14)	0.21	(0.13)	0.23	(0.12)			0.30*	(0.15)	0.28	(0.14)	0.31	(0.13)
Gender			0.05	(0.14)	0.10	(0.13)	0.18	(0.13)			0.13	(0.15)	0.15	(0.15)	0.25	(0.14)
AME-ORS^a^					− 0.04	(0.14)	0.06	(0.13)					− 0.09	(0.15)	0.03	(0.14)
AME-*EM*^b^					0.42**	(0.13)	0.51**	(0.13)					0.34**	(0.15)	0.43**	(0.14)
AME-ORS × AME-EM							0.33**	(0.12)							0.40**	(0.13)
Random effects																
Level 1 intercept variance (day level)	0.59		0.59		0.59		0.59		0.99		0.99		0.99		0.99	
Level 2 intercept variance (person level)	0.84		0.82		0.66		0.57		0.91		0.83		0.73		0.60	
– 2 × log (lh)	616.6		613.0		602.0		594.2		711.7		705.8		699.2		689.9	
Δ – 2 × log (lh)			3.3		11.0**		7.8**				5.9*		6.6*		9.3**	
*df*			2		2		1				2		2		1	
*R*^2^ (person level)			0.024		0.214		0.321				0.088		0.198		0.341	
Δ *R*^2^ (person level)					0.190		0.107						0.100		0.143	

Gender, age, AME-ORS, and AME-EM are person-level (Level 2) variables; work engagement and flow experience are day-level variables (Level 1). The R-squared values for the day level are not reported since the value is constant between models

^*^p < .05; **p < .01

^a^Achievement motive enactment via the object recognition system

^b^Achievement motive enactment via extension memory

Hypothesis 2 proposed that achievement motive enactment via the object recognition system would be negatively related to (a) day-specific work engagement and (b) day-specific flow experience. However, contrary to this proposition, multilevel estimates revealed that achievement motive enactment via the object recognition system did not significantly predict either work engagement (γ = − 0.04, SE = 0.14, *n.s.*) or flow experience (γ = − 0.09, SE = 0.15, *n.s.*).

In Hypothesis 3, we predicted that achievement motive enactment via extension memory would interact with achievement motive enactment via the object recognition system in predicting (a) work engagement and (b) flow experience such that the presence of both enactment types would be most adaptive for self-regulation. In line with our prediction, multilevel estimates revealed that achievement motive enactment via extension memory and achievement motive enactment via the object recognition system significantly interacted to predict both work engagement (γ = 0.33, SE = 0.12, *p* < 0.05) and flow experience (γ = 0.34, SE = 0.15, *p* < 0.01). Model 3 showed an improved fit compared with that of Model 2, as indicated by the difference in the log likelihood ratios for work engagement (Δ − 2 × log = 7.8, df = 1, *p* < 0.01) and flow experience (Δ − 2 × log = 9.3, df = 1, *p* < 0.01). To facilitate the interpretation of the interactions, we depicted the interactions and performed simple slope tests, as recommended by Preacher et al. (2006). As Appendix Fig. 1 shows, the interaction patterns are consistent with Hypothesis 3. In particular, for people high in achievement motive enactment via extension memory, the relationships between achievement motive enactment via the object recognition system and day-specific (a) work engagement and (b) flow experience were more positive (γ = 0.39, t = 3.10, *p* < 0.01; γ = 0.43, t = 3.37, *p* < 0.01, respectively) than for those low in achievement motive enactment via extension memory (γ = − 0.28, t = 1.78, *p* < 0.10; γ = − 0.37, t = 2.36, *p* < 0.05, respectively).

**Fig. 1:**
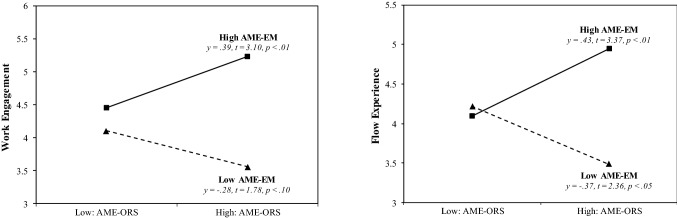
Interaction plots for predicting work engagement and flow experience. *AME-ORS* achievement motive enactment via the object recognitions system, *AME-EM* achievement motive enactment via extension memory

### Additional analyses

We further analyzed whether the effects of the two-way interaction on flow and work engagement changed over time. After extracting the individual slopes for the study day as a predictor of flow and work engagement, we tested whether the two-way interaction had a significant effect on the slopes. For both outcomes, the interaction was not significant (flow experience: γ = 0.04, SE = 0.03, *n.s.;* work engagement: γ = 0.03, SE = 0.02, *n.s.*). This finding indicates that the interaction effect is stable over time, at least over the course of the study.

In addition, we tested whether the two-way interaction would predict the variance in both outcomes after the study day was controlled. The results indicated that the day did not have an influence on the variance for either flow or work engagement (γ =  − 0.08, SE = 0.04, *n.s.;* γ =  − 0.01, SE = 0.03, *n.s.*, respectively*)*. The interaction remained significant (γ = 0.29, SE = 0.09, *p* < 0.01; γ = 0.15, SE = 0.06, *p* < 0.05, respectively), signifying that even the study day was controlled, flow and work engagement were more pronounced under favorable conditions.

## Discussion

We conducted a diary study to examine the main and interaction effects of achievement motive enactment via the object recognition system and extension memory on day-specific flow and work engagement. Our findings indicate that achievement motive enactment via extension memory is positively related to both work engagement and flow experience. Specifically, the more strongly individuals enact their achievement motive via extension memory, the higher their overall level of day-specific work engagement and flow experiences. This finding indicates that individuals with sufficient access to their extended networks of individual prior experiences and self-representations experience higher levels of flow and work engagement at work. The predicted negative impact of achievement motive enactment via the object recognition system on flow and work engagement, however, could not be empirically supported. Thus, the achievement motive enactment via the object recognition system has no adverse impact on flow and work engagement at work. Moreover, we examined the interaction effects between achievement motive enactment via extension memory and achievement motive enactment via the object recognition system in the prediction of flow and work engagement. In line with previous studies investigating the dynamic interplay of the macrosystems postulated in PSI theory (e.g., Bledow et al., 2011; Yang et al., 2016), we found that the presence of both dispositional forms of enactment is most beneficial for flow and work engagement.

## Study 2

Prior studies have shown that approximately 55–60% of the variance in flow (Diestel et al., 2015; Rivkin et al., 2018) and approximately 60–65% of the variance in work engagement (Sonnentag, 2003; Venz et al., 2018) can be explained by between-person variance, meaning variations that are caused by differences between persons. Accordingly, we reported 48% (flow experience) and 59% (work engagement) between-person variance in study 1, indicating stable patterns of flow experience and work engagement that consequently must be able to be predicted by level 2 variables. Even in cases of strong intraindividual variations over the course of a study, there are factors that are stable over time in predicting both outcomes. Differences between persons can be explained by differences in personality (traits), job characteristics, or their interaction. In this vein, van den Berg and Feij (2003) demonstrated that personality traits and job characteristics can have additive and nonadditive effects on behavioral outcomes. Our results from study 1 suggest that extension memory contributes to an emotional and holistic way of experiencing good access to stored experiential knowledge. This experience should be especially adaptive in situations when a large amount of information and contradictory or ambiguous elements require parallel processing (Scheffer & Manke, 2018). In work environments, individuals increasingly face ambiguous and unclear task requirements as organizations become more flexible and dynamic and establish new or expanded roles (Schmidt et al., 2014). Kahn (1990) identified role clarity as a resource that facilitates work engagement. In addition, Lang et al. (2007) demonstrated that role clarity can buffer the deleterious effects of job stressors on well-being since perceived clarity increases the likelihood of attaining one’s personally valued goals (Bliese & Castro, 2000). Prior studies have demonstrated direct (Seppälä et al., 2015) and indirect (Halbesleben, 2010) links of role clarity to engagement at work.

### The impact of role clarity on achievement motive enactment

We propose that role clarity buffers the interaction of different forms of achievement motive enactment. Our propositions are derived from two lines of argument: the plasticity hypothesis and research on selective optimization and compensation (SOC) strategies on role clarity and work engagement. The plasticity hypothesis (Brockner, 1983) states that individuals are influenced by environmental factors to different degrees according to their individual characteristics. Prior research has revealed that employees with low self-esteem are more susceptible to work environment factors (e.g., role ambiguity) than their counterparts with high self-esteem (Ganster & Shaubroeck, 1991; Jex & Elacqua, 1999; Mossholder et al., 1981; Pierce et al., 1993). A similar pattern was found by Trépanier et al. (2013) and Gillet et al. (2016), who reported that the effects of motivation (free volitional choice vs. internal and/or external pressures) on anxiety and distress were moderated by role clarity.

Prior research on SOC also suggests that the impact of role clarity on work engagement is moderated by resources that are linked to adaptiveness in adverse situations (Zacher & Frese, 2011). According to Venz et al. (2018), SOC compensates for low role clarity. Conversely, when the structure of a task is transparent and role clarity is given, there is no need for adaptive self-management. This finding indicates that role clarity makes the structure of the task clear and conveys meaningfulness.

According to our lines of reasoning, we expect role clarity to moderate the interaction between achievement motive enactment via the object recognition system and via extension memory. When role clarity is low, the conjunction of both forms of achievement motive enactment (via the object recognition system and extension memory) should exert beneficial impacts on flow and work engagement. In this case, extension memory represents a protective mechanism that supports individuals in adjusting their goals to the current situation and optimizing the investment of available resources. Without this integrative function, individuals are likely to experience a mismatch between their skills and the challenge of a task (Venz et al., 2018). When there is a high level of clarity regarding task procedures, role conditions and goal achievement, motivational processes have less influence on flow and work engagement, as the match between the skills of the individual and the challenge of the task is enabled by the clear structure of the task (Bliese & Castro, 2000; Lang et al., 2007).

#### H4:

Three-way interaction: Role clarity moderates the interaction effect of achievement motive enactment via extension memory and achievement motive enactment via the object recognition system on (a) work engagement and (b) flow experience. In cases of low role clarity, the conjunction of achievement motive enactment via extension memory and achievement motive enactment via the object recognition system exerts beneficial effects on work engagement and flow experience, whereas in cases of high role clarity, neither form of achievement enactment interacts in predicting the two outcome variables.

### Methods

#### Sample and study design

The procedure for recruiting the participants and completing the diary study was the same as that in study 1, with the only difference being that the study was conducted over 10 (instead of 5) consecutive workdays. Again, we ideally asked people who were employed in the services sector and who had daily work-related contact with clients, patients, or customers. In total, we recruited 223 people (response rate: 84.84%; 1892 daily measurement points). In contrast to that for study 1, the data collection for study 2 took place during the COVID-19 pandemic between April and May 2020. The first study was conducted before the pandemic and its far-reaching impacts, but it is important to note that work conditions (i.e., role clarity) in study 2 played a more prominent role in the outcome variables flow and work engagement. It is likely that the means of and variance in role clarity differed from what would have been measured before the outbreak. The percentage of home workers within study 1 was not recorded but given that we recruited individuals from the service sector, it is likely that the rate was fairly low. For study 2, 73.04% of participants worked exclusively from home during the data collection period. Before the COVID-19 outbreak, the share of teleworking hours among the participants’ total working hours was 16.14% (SD = 26.8).

#### Measures

We assessed achievement motive enactment, work engagement, and flow experience with the same scales from study 1.

##### Role clarity

Role clarity was measured using the Role Ambiguity Scale (Sodenkamp & Schmidt, 2000), which contains subdimensions for the clarity of work methods (e.g., “In my job, I know exactly how to proceed in order to do my job well.”) and clarity of work processes (e.g., “In my job, I know exactly when to do a particular task.”). The items are scored on a seven-point rating scale ranging from 1 (does not apply) to 7 (completely applies).

##### Control variables

Previous research indicated that high stress impedes flow and work engagement (e.g. Peifer et al., 2014). The COVID-19 pandemic may have affected people in different ways. Therefore, we added emotional exhaustion as the focal dimension of burnout as a control variable. Emotional exhaustion was measured with eight items from the German translation (Büssing & Perrar, 1992) of the Maslach Burnout Inventory (Maslach et al., 1996). An exemplary item is “I feel emotionally drained by my work”. The items are scored on a six-point rating scale ranging from 1 (never) to 6 (very often).

##### Construct validity

As in study 1, we conducted multilevel confirmatory factor analyses (MCFAs) to test the divergent validity of the day-level variables work engagement and flow experience. First, we tested a two-factor measurement model including the two variables as distinct factors. The fit indices for this model indicated a satisfactory fit: χ2 (128) = 211.38, *p* < 0.01, root mean square error of approximation (RMSEA) = 0.054, comparative fit index (CFI) = 0.957, standardized root mean square residual within-person/between-person (SRMRw/SRMRb) = 0.044/0.046. In contrast, a model integrating the two day-level variables into one common factor performed worse (χ2 (130) = 1223.79, *p* < 0.01, RMSEA = 0.067, CFI = 0.910, SRMRw/SRMRb = 0.040/0.041).

Taken together, the results of the conducted MCFSs suggest that the two day-level variables work engagement and flow experience represent distinct constructs.

## Results

Appendix Table 3 displays the descriptive statistics, internal consistencies (Cronbach’s alphas) and correlations among the study variables. The analyses of variance suggest substantial Level 1 variance in the outcomes (work engagement: 47.5%; flow experience: 43.9%).

**Table 3:** Means, standard deviations, internal consistencies (Cronbach’s Alphas) and intercorrelations of the study variables

Variable	1	2	3	4	5	6	7	8
1. Work engagement	(0.96)	**0.84**						
2. Flow experience	**0.89**	(0.90)						
3. AME-ORS^a^	**− 0.24**	**− 0.15**	(0.84)					
4. AME-EM^b^	**0.43**	**0.37**	**− 0.39**	(0.67)				
5. Role clarity	0.09	0.05	**− **0.03	0.07	(0.91)			
6. Emotional exhaustion	**− 0.24**	**− 0.13**	**0.34**	**− 0.34**	**− 0.19**	(0.87)		
7. Age	0.10	0.08	**− 0.20**	0.09	**0.15**	**− **0.06	–	
8. Gender^c^	**− **0.01	**− **0.02	**− 0.18**	**− **0.01	**− 0.15**	**− **0.01	0.02	–
M	4.11	4.15	1.78	3.03	4.33	2.60	38.65	1.44
SD	1.00	1.04	0.6	0.43	1.19	0.96	11.31	0.51

The Cronbach’s alpha for day-level variables is the mean internal consistency averaged over all measurement days. Correlations below the diagonal are person-level correlations (*N* = 230); those above the diagonal are day-level

correlations (*N* = 1892). Numbers in bold *p* < .05

^a^Achievement motive enactment via the object recognition system

^b^Achievement motive enactment via extension memory

^c^Gender (1 = female, 2 = male)

### Hypothesis testing

We tested our hypotheses by comparing four different models. In the null model, we included the intercept as the only predictor. In Model 1, we added the control variables age, gender, and emotional exhaustion. In Model 2, we entered achievement motive enactment via the object recognition system, achievement motive enactment via extension memory, and role clarity at Level 2. In Model 3, we added the two-way interactions between the variables introduced in Model 2. Finally, in Model 4, we introduced the three-way interaction achievement motive enactment via the object recognition × achievement motive enactment via extension memory × role clarity.

In Hypothesis 4, we predicted a three-way interaction between achievement motive enactment via extension memory, achievement motive enactment via the object recognition system, and role clarity in predicting (a) work engagement and (b) flow experience. In line with our prediction, multilevel estimates revealed that the variables significantly interacted to predict work engagement (γ =  − 0.17, SE = 0.06, *p* < 0.01; see Appendix Table 4) and flow experience (γ =  − 0.19, SE = 0.07, *p* < 0.01; see Appendix Table 5). Model 4 showed an improved fit compared with that of Model 3, as indicated by the difference in the log likelihood ratios for work engagement (Δ – 2 × log = 3.5, df = 1, *p* < 0.01) and flow experience (Δ – 2 × log = 3.9, df = 1, *p* < 0.01). To facilitate the interpretation of the interactions, we depicted the interactions and performed simple slope tests, as recommended by Preacher et al. (2006). As Appendix Figs. 2 (work engagement) and 3 (flow experience) show, the interaction patterns are consistent with Hypothesis 4. In particular, the moderating effect of achievement motive enactment via extension memory on flow experience is stronger in cases of low role clarity (γ = 0.39, t = 2.93, *p* < 0.05) than in cases of high role clarity.

**Table 4:** Multilevel estimates for the prediction of work engagement

Effects	Work engagement									
	Null model		Model 1		Model 2		Model 3		Model 4	
	γ	SE	γ	SE	γ	SE	γ	SE	γ	SE
Fixed effects										
Intercept	4.11**	(0.07)	4.12**	(0.20)	4.09**	(0.19)	4.06**	(0.19)	4.14**	(0.19)
Age			− 0.01	(0.13)	− 0.00	(0.12)	− 0.00	(0.12)	− 0.03	(0.12)
Gender			0.08	(0.06)	0.04	(0.06)	0.03	(0.06)	0.03	(0.06)
Emotional exhaustion			− 0.24**	(0.07)	− 0.09	(0.07)	− 0.09	(0.07)	− 0.11	(0.07)
AME-ORS^*a*^					− 0.05	(0.07)	− 0.06	(0.07)	− 0.03	(0.07)
AME-EM^b^					0.38**	(0.07)	0.39**	(0.07)	0.38**	(0.07)
Role clarity (RC)					0.05	(0.07)	0.03	(0.07)	− 0.05	(0.07)
AME-ORS × AME-EM							− 0.05	(0.05)	0.06	(0.07)
AME-ORS × RC							− 0.03	(0.07)	− 0.02	(0.07)
AME-EM × RC							0.03	(0.07)	0.04	(0.07)
AME-ORS × AME-EM × RC									− 0.17**	(0.06)
Random effects										
Level 1 intercept variance (day level)	0.94		0.94		0.94		0.94		0.94	
Level 2 intercept variance (person level)	0.85		0.80		0.68		0.69		0.66	
− 2 × log (lh)	− 2862.8		− 2855.6		− 2838.0		− 2837.3		− 2833.8	
Δ − 2 × log (lh)			7.2**		17.6**		0.7		3.5**	
*df*			3		3		3		1	
*R*^2^ (person level)			0.059		0.200		0.188		0.224	
Δ *R*^2^ (person level)					0.141		− .0.012		0.036	

Gender, age, emotional exhaustion, AME-ORS, AME-EM, and role clarity are person-level (Level 2) variables; work engagement is a day-level variable (Level 1). The R-squared values for the day level are not reported since the value is constant between models

^*^p < .05; **p < .01

^a^Achievement motive enactment via the object recognition system

^b^Achievement motive enactment via extension memory

**Table 5:** Multilevel estimates for the prediction of flow experience

Effects	Flow experience									
	Null model		Model 1		Model 2		Model 3		Model 4	
	γ	SE	γ	SE	γ	SE	γ	SE	γ	SE
Fixed effects										
Intercept	4.15**	(0.07)	4.18**	(0.21)	4.13**	(0.20)	4.12**	(0.21)	4.21**	(0.20)
Age			− 0.02	(0.14)	− 0.01	(0.13)	− 0.01	(0.13)	− 0.04	(0.13)
Gender			0.08	(0.07)	0.05	(0.07)	0.05	(0.07)	0.04	(0.07)
Emotional exhaustion			− 0.13**	(0.07)	− 0.01	(0.07)	− 0.01	(0.08)	− 0.03	(0.08)
AME-ORS^a^					0.01	(0.08)	0.01	(0.08)	0.04	(0.08)
AME-EM^b^					0.38**	(0.07)	0.38**	(0.08)	0.37**	(0.08)
Role clarity (RC)					0.02	(0.07)	0.00	(0.08)	− 0.08	(0.08)
AME-ORS × AME-EM							− 0.01	(0.06)	0.11	(0.07)
AME-ORS × RC							− 0.06	(0.07)	− 0.05	(0.07)
AME-EM × RC							− 0.00	(0.08)	0.01	(0.08)
AME-ORS × AME-EM × RC									− 0.19**	(0.07)
Random effects										
Level 1 intercept variance (day level)	1.15		1.15		1.15		1.15		1.15	
Level 2 intercept variance (person level)	0.90		0.89		0.79		0.80		0.77	
− 2 × log (lh)	− 3039.3		− 3036.8		− 3023.4		− 3023.0		− 3019.1	
Δ − 2 × log (lh)			2.5		13.4**		0.4		3.9**	
*df*			3		3		3		1	
*R*^2^ (person level)			0.011		0.122		0.111		0.144	
Δ *R*^2^ (person level)					0.111		− 0.011		0.033	

Gender, age, emotional exhaustion, AME-ORS, AME-EM, and role clarity are person-level (Level 2) variables; flow experience is a day-level variable (Level 1). The R-squared values for the day level are not reported since the value is constant between models

^*^p < .05; **p < .01

^a^Achievement motive enactment via the object recognition system

^b^Achievement motive enactment via extension memory

**Fig. 2:**
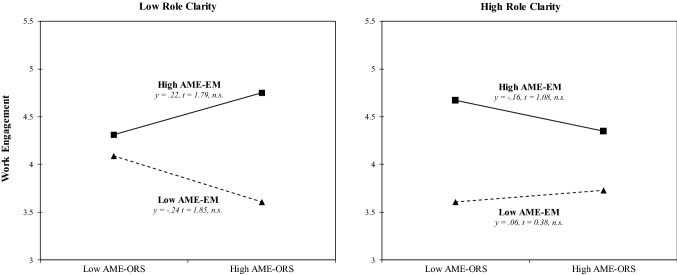
Three-way interaction plots for predicting work engagement. *AME-ORS* achievement motive enactment via the object recognitions system, AME-EM achievement motive enactment via extension memory

**Fig. 3:**
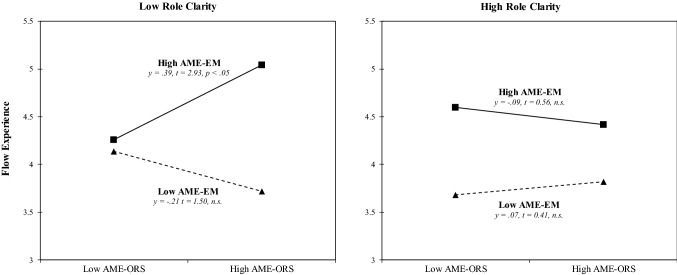
Three-way interaction plots for predicting flow experience. *AME-ORS* achievement motive enactment via the object recognitions system, *AME-EM* achievement motive enactment via extension memory

## General discussion

Theoretical insights in positive psychology mainly draw from a large body of empirical evidence on flow and work engagement as fluctuating states and environmental conditions conducive to these states. When looking at dispositional precursors, research is mostly concerned with personality traits (e.g., conscientiousness). However, much less is known about how dispositional motives and their enactment shape both outcomes. In addressing this issue, we sought to identify how two forms of achievement motive enactment (via the object recognition system and via extension memory) interact with each other. PSI theory suggests that simultaneous access to both macrosystems is beneficial for flow and work engagement over time. In study 1, our results indicated that the conjunction of both dispositional forms of enactment exerts beneficial effects on general levels of day-specific flow experience and work engagement. In study 2, we tested whether role clarity moderated the interaction of both achievement motive enactment types. In support of our predictions, we found that when role clarity was low (i.e., high role ambiguity), simultaneous access to both macrosystems via both forms of achievement motive enactment interacted to predict flow experience and work engagement. In contrast, in cases of high role clarity (i.e., low role ambiguity), the simultaneous enactment of achievement motive via both macrosystems did not predict both flow and work engagement over time.

## Theoretical implications

Our research offers at least four implications for the literature on achievement motive enactment, day-specific work engagement, and flow experience. First, we not only contribute insights about motivation (what people strive for) and volition (how people strive) but also integrate both perspectives by examining interaction effects between motivation and volition. In doing so, we reveal that the object recognition system, a macrosystem that is linked to the presence of negative affect, can be even beneficial for flow and work engagement when both the object recognition system and extension memory are activated during the enactment of an achievement motive. Going beyond existing knowledge about affect modulation, according to which negative affect can even be beneficial for work engagement and task performance (e.g., Bledow et al., 2011; Yang et al., 2016), we provide more nuanced insights into the underlying mechanisms of personality systems interactions. On the one hand, when individuals deal with challenges or problems at work, important cues and information are provided through the stimulation of detailed object-related information processing. On the other hand, strong task-focused, goal-directed regulation of attention, behavior, and decision-making processes are induced by repeatedly occurring negative affect, which can prevent obstacles to task completion and goal achievement.

Second, we advance empirical evidence on PSI theory by differentiating individual achievement motive via the two macrosystems, i.e., extension memory and object recognition system. If individuals enact their achievement motive via extension memory, they experience higher levels of day-specific flow and work engagement. Achievement motive enactment via the object recognition system, which causes employees to focus on potential hindrances and difficulties, can be an asset when employees also draw from their extension memory.

Third, the present study contributes to the understanding of the impact of achievement motive on flow and work engagement by examining two forms of achievement motive enactment. In line with previous research on individual differences and flow and work engagement (e.g., Kahn, 1990), we found that achievement motive enactment shapes people’s tendencies regarding the frequency of and ability for flow and work engagement. The reported main and interaction effects represent unique insights into the dispositional antecedents of flow and work engagement that have been neglected in motivational research. More precisely, the demonstrated main effects indicate that achievement motive enactment can vary in its adaptiveness for flow and work engagement since the way the achievement motive is fulfilled differs. The interaction effect additionally indicates that simultaneous access to both forms of enactment is most beneficial for flow and work engagement, as it facilitates self-development. By exploring both underlying macrosystems in terms of how achievement motive shapes both motivational outcomes, our findings add to existing knowledge about affective shifts (Bledow et al., 2011; Yang et al., 2016). Whereas past research has proposed changes in affect to be indicators of a dynamic interplay between object recognition systems and extension memory, the present interaction patterns show how stable and dispositional tendencies in both underlying systems influence the impact of an important motivational driver on flow and work engagement, thereby explaining how and why achievement motives facilitate both outcomes.

Fourth, our results underline the importance of role clarity in the relation between achievement motive enactment and flow and work engagement. In line with the interaction of adaptive strategies and role clarity on work engagement reported by Venz et al. (2018), the integrative function of extension memory is necessary only when task procedures, role conditions and goal achievement are not clear. If they are clear, motivational processes have less influence on flow and work engagement, as a match between the individual’s skills and the challenge of the task is enabled by the clear structure of the task (Bliese & Castro, 2000; Lang et al., 2007). Whereas the data for study 1 were collected before the COVID-19 outbreak, the data for our second study were collected in April and May 2020, just after a national lockdown for crisis prevention in Germany was announced at the end of March. During that time, both employees and employers experienced many ambiguities regarding their tasks, responsibilities, futures, and other important facets of work. Clarity about work tasks and processes therefore played a crucial role in motivational states such as flow and work engagement.

## Limitations and suggestions for future research

Despite its several contributions, our study is not without limitations. First, all study variables were operationalized on the basis of self-report questionnaires, which imply the risk of common method bias (Podsakoff et al., 2003). However, in line with Siemsen et al. (2010), the effects of the current study likely reflect valid relations rather than common method artifacts since a high common method variance reduces the probability of detecting interaction effects. Nonetheless, future studies would benefit from different operationalizations of achievement motive enactment, such as neurophysiological measures (e.g., hemispheric laterality; Kuhl & Kazén, 2008).

Second, the drop-out rate in our first sample was approximately 28%, indicating that participants, on average, completed only approximately 3.5 out of 5 day-specific questionnaires. This could be seen as an indication of the low conscientiousness of some participants, which could have had an influence on the results. However, there was no deterioration of compliance over time, which indicates no statistical influence of the drop-out rate.

Third, our correlational data structure does not permit strong causal conclusions. However, the measurement of dependent and independent variables at separate times allows for more causal conclusions than a simple cross-sectional study at a single time (cf. Aguinis et al., 2013).

Fourth, based on our findings that forms of achievement motive enactment shape how employees experience flow and work engagement, we encourage scholars to examine the extent to which these dispositional antecedents are related to day-specific affects and their shifts (cf. Bledow et al, 2011). For example, as achievement motive enactment via extension memory represents a general ability, it is likely to interfere with affective shifts on a daily basis. Another interesting goal for future studies could be to examine whether the forms of achievement motive enactment via the two other macrosystems postulated in PSI theory (intuitive behavior control and intention memory; Kuhl, 2000) are also dispositional antecedents for flow and work engagement.

Fifth, our study focused on achievement motive given its strong link to flow and work engagement (e.g., autotelic personalities; Csikszentmihalyi et al., 1993). However, not all work behavior is solely goal-related and reflected in the achievement domain. Future research might consider the (moderating or mediating) impact of power and affiliation motive (cf. Mc Clelland, 1985).

## Conclusion

Motive dispositions are important precursors of daily flow and work engagement. Whereas enacting the achievement motive with all of our experiential and integrative capacity (extension memory) is beneficial, achievement motive enactment with an alert-focus on finding negative aspects in an overall positive context (object recognition) is detrimental. However, people who combine these opposing enactment types in their personality show highest levels of flow and work engagement on a daily basis. This has practical implications for personnel selection and development. On one hand, companies may benefit from focusing enactment types when identifying potential candidates for job positions that require high achievement motives. On the other hand, personnel development can enhance integrative competencies, thereby facilitating enactment via extension memory.

## Appendix

See Figs. 1, 2 and 3 and Tables 1, 2, 3, 4 and 5.
